# Virome of wild rats (*Rattus norvegicus*) captured far from pig farms in Jiangsu province of China reveals novel porcine circovirus type 2d (PCV2d) sequences

**DOI:** 10.1186/s12985-023-02005-2

**Published:** 2023-03-09

**Authors:** Min Zhao, Siwen Bao, Diandian Xu, Jingxian He, Han Zhang, Likai Ji, Shixing Yang, Xiaochun Wang, Quan Shen, Jia Liu, Qing Zhang, Xiao Ma, Wen Zhang, Tongling Shan

**Affiliations:** 1 Department of Microbiology, School of Medicine, Jiangsu University, Zhenjiang, 212003 Jiangsu China; 2 Shanghai Veterinary Research Institute, Chinese Academy of Agricultural Sciences, Shanghai, 200241 China; 3 Qinghai Institute of Endemic Disease Prevention and Control, Xining, 811602 Qinghai China; 4 School of Medicine, Suzhou University, Suzhou, 215031 Jiangsu China

**Keywords:** PCV2, Wild rat, Phylogenetic analysis

## Abstract

**Background:**

Porcine circovirus type 2 (PCV2) has caused great economic losses in the global pig industry. There have been published records of wild rats acting as the reservoirs of PCV2 (only PCV2a and PCV2b), but almost all of which were related to the PCV2-infected swine herds.

**Results:**

In this study, we carried out the detection, amplification, and characterization of novel PCV2 strains in wild rats that were captured far from pig farms. Nested PCR assay demonstrated that the kidney, heart, lung, liver, pancreas, and large and small intestines of rats were screened positive for PCV2. We subsequently sequenced two full genomes of PCV2 in positive sample pools, designated as js2021-Rt001 and js2021-Rt002. Genome sequence analysis indicated that they had the highest similarity to nucleotide sequences of porcine-origin PCV2 isolates in Vietnam. Phylogenetically, js2021-Rt001 and js2021-Rt002 were a part of the PCV2d genotype cluster, which is a predominant genotype circulating worldwide in recent years. The antibody recognition regions, immunodominant decoy epitope, and heparin sulfate binding motif of the two complete genome sequences coincided with those previously reported.

**Conclusions:**

Our research reported the genomic characterization of two novel PCV2 strains (js2021-Rt001 and js2021-Rt002) and provided the first supported evidence that PCV2d could naturally infect wild rats in China. However, whether the newly identified strains have potential for circulating in nature in vertical and horizontal transmission or inter-species jumping between rats and pigs needs further research.

**Supplementary Information:**

The online version contains supplementary material available at 10.1186/s12985-023-02005-2.

## Background

Porcine circovirus type 2 (PCV2) is a small, non-enveloped, single-strand circular DNA (ssDNA) virus with 1766–1768 nucleotides (nt) in length, classified under the genus *Circovirus* in the family *Circoviridae* [1]. PCV2 was first isolated from tissues of pigs in western Canada in 1998, many more PCV2 isolates have been reported worldwide since then, posing a continuing threat to veterinary public health [2]. PCV2 can cause a group of diverse multi-factorial syndromes in domestic pigs and wild boars across the globe, collectively named PCV-associated diseases (PCVADs), such as post-weaning multisystemic wasting syndrome (PMWS), porcine dermatitis and nephropathy syndrome (PDNS), porcine respiratory disease complex (PRDC), enteritic disease, and reproductive failure [3–6].

PCV2 is known for its high rates of infection, transmission, and mutation together with inter- and intra-genotype recombination, which is considered to be an important evolutionary mechanism for the emergence of new genotypes [7–10]. Compared with other DNA viruses, PCV2 has a higher evolutionary rate (1.21 × 10^−3^ to 6.57 × 10^−3^ substitutions/site/year) [4, 11]. Currently, PCV2 has been classified into eight genotypes, PCV2a to PCV2e, and the newly reported PCV2f, PCV2g, and PCV2h [12, 13], of which only three genotypes (PCV2a, PCV2b, and PCV2d) have a persistent and broad worldwide distribution, especially in pig-producing countries, causing significant economic losses and veterinary public health issues [12, 14–16].

Domestic pigs and wild boars are generally considered as the natural reservoirs of PCV2. But currently, the known host range of this virus has expanded to humans [17] and other non-porcine mammals (such as bovids, minks, foxes, dogs, raccoon dogs, goats, rats, and mice) [18–25], making it more conducive to virus transmission and prevalence. Experimental mice are generally used as the model to investigate the role of rodents in carrying, replicating, and transmitting PCV2 [19, 26–29]. It has been reported that PCV2 (genotypes PCV2a and PCV2b) can frequently spillover from pigs to rodents on pig farms [20, 30, 31]. However, there was no report on the presence of the currently predominant genotype PCV2d in wild rats and PCV2 infection in rats outside pig farms. In this study, two PCV2d strains were identified from wild rats (*Rattus norvegicus*) captured far from pig farms in Jiangsu province, China. This finding provided the first evidence that genotype PCV2d has the capacity to naturally infect rats.

## Methods

### Sample collection, library construction, and next-generation sequencing

In June 2021, a total of 14 tissue samples from two wild rats identified as *Rattus norvegicus* based on the mitochondrial 12S rRNA and 16S rRNA genes were collected in Jiangsu province. Here the tissue samples were treated as described in our previous research [32] in a biosafety level 2 facility according to strict operating procedures to avoid possible laboratory environment, reagent, and cross-sample contamination. All nucleic acid samples from the same individual were combined into one pool. The total nucleic acid was extracted using QIAamp Viral RNA Mini Kit (QIAGEN) according to the manufacturer’s protocol. Briefly, the nucleic acid sample pools were used for viral metagenomic library construction as described in our previously published papers [32–34]. To exclude the possibility of cross-library contamination, a blank control, sterile ddH2O (Sangon, Shanghai, China) was prepared and going through the entire library preparation process. Afterward, two rat libraries along with a control library were sequenced on the Illumina NovaSeq 6000 platform with 250 base paired-end reads with dual barcoding.

### Bioinformatic analyses

For bioinformatics analyses, the generated reads were debarcoded using vendor software from Illumina. Clonal reads were removed, and low sequencing quality tails were trimmed using Phred quality score 30 (Q30) as the threshold. The cleaned reads were de novo assembly using the Geneious Prime (v2019.2.3) [35]. To find viral-related sequences, the assembled contigs and singlet sequences were then matched against the NCBI non-redundant nucleotide (NT) and protein (NR) databases using BLAST (E-value < 10^−5^) [36]. Candidate viral hits were then compared to a non-virus non-redundant protein database to remove false positive viral hits.

### PCR detection and amplification of the whole genome of PCV2

We designed nested PCR (nPCR) primers for PCV2 screening and full-genome acquisition based on the assembled PCV2-related contigs and the best hits of them to nucleotide sequences in the NCBI database. Three sets of specific nPCR primers were used to generate three overlapping fragments. Primers used in this study are listed in Table 1. The nPCR conditions are as follows: 95 °C for 5 min for initial denaturation, 31 cycles of denaturation at 95 °C for 30 s, annealing at 52 °C (first round) or 60 °C (second round) for 30 s, and elongation at 72 °C for 40 s, ended with a final elongation at 72 °C for 5 min. PCR products of fragments were purified with MiniBEST Agarose Gel DNA Extraction Kit (TakaRa, Dalian, China), subcloned into the plasmid pMD™-18T vector (TaKaRa, Dalian, China), and subsequently transformed into competent *Escherichia coli* DH5α cells (TaKaRa, Dalia, China). At least three positive clones of each fragment were sent to Sangon Biotech for Sanger sequencing. Subsequently, the sequencing data were reassembled to generate the complete genomes of PCV2 in Geneious Prime.

**Table 1:** The primers of nested PCR used for detection and amplification of the PCV2 genome

Fragment	Primer name	Application	Primer sequence (5′–3′)
1	1WF/1NF	1st and 2nd round	TGCTGTGAGTACCTTGCTGG
1	1WR	1st round	CCGTGGATTGTTCTGTAGCA
1	1NR	2nd round	GTAGATCATCCCAGGGCAGC
2	2WF^a^	1st round	TGCTGTGAGTACCTTGCTGG
2	2WR^a^	1st round	CCATCTTGGCCAGATCCTCC
2	2NF^a^	2nd round	GCAGACCCGGAAACCACATA
2	2NR^a^	2nd round	GAATGTGGACATGATGAGAT
3	3WF	1st round	GGGTTATGGTATGGCGGGAG
3	3WR	1st round	CAAACGTTACAGGGTGCTGC
3	3NF	2nd round	ATAACAGCAGTGGAGCCCAC
3	3NR	2nd round	CCAGCAAGGTACTCACAGCA

^a^The primers were also used to detect the tissue distribution of PCV2 in wild rats

### Phylogeny of viruses and data analysis

All genome and protein sequence alignments were performed using ClustalW in MEGA11 (v11.0.11) [37] with the default settings. The phylogenetic tree of complete genome nucleotide sequences was constructed using the Maximum-likelihood (ML) method in MEGA11 with 1000 bootstrap replicates under the TN93 substitution model and gamma-distributed with invariant sites (G + I). The phylogenetic tree of PCV2 ORF2 genes was generated with the best-fit TN93 + G nucleotide substitution model. Multiple sequence alignment of ORF2-encoded Cap protein amino acid (aa) sequences of PCV2d strains were visualized with JALVIEW (v2.11.2.2) [38].

## Results

### Virome analysis and identification of rat-associated PCV2

**Fig. 1:**
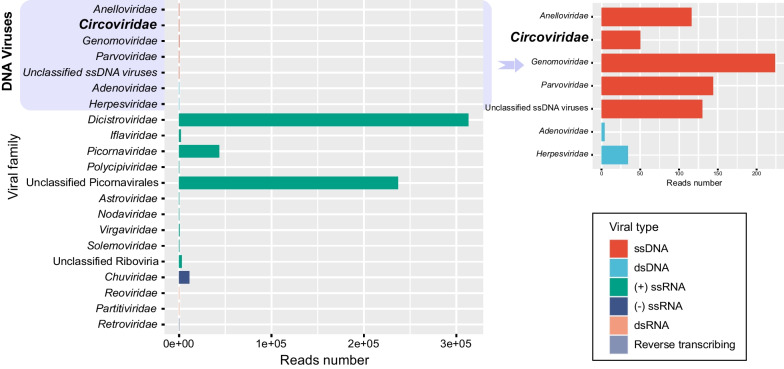
Bar plots showing taxonomic category and viral abundance in pooled rat tissue samples

All rat tissue samples were divided into two pools/libraries (Rt001 and Rt002) for next-generation sequencing (NGS), generating a total of 4,309,426 reads, among which 14.20% (n = 611,831) reads showed similarity to known eukaryotic viruses. The remaining 85.80% (n = 3,697,595) of sequencing data aligned to eukaryotes or prokaryotes, bacteriophages and those with no significant similarity to any aa sequence in the NR database. The blank control library generated a small number of raw reads (n = 13,668) which were free of viral sequences. At the family level, eukaryotic viral reads were classified into two families of double-stranded DNA viruses (dsDNA virus: *Adenoviridae* and *Herpesviridae*), four families of single-stranded DNA viruses (ssDNA virus: *Anelloviridae*, *Circoviridae*, *Genomoviridae*, and *Parvoviridae*), two families of double-stranded RNA viruses (dsRNA virus: *Reoviridae* and *Partitiviridae*), nine families of single-stranded RNA viruses (ssRNA virus: *Astroviridae*, *Chuviridae*, *Nodaviridae*, *Dicistroviridae*, *Iflaviridae*, *Picornaviridae*, *Polycipiviridae*, *Virgaviridae*, and *Solemoviridae*), *Retroviridae* family and unassigned viruses (Fig. 1). Compared to RNA viruses, a relatively small number of reads (n = 702) were identified as being homologous to DNA viruses. In particular, BLASTx analysis revealed two contigs assembled from the reads in the family *Circoviridae* of Rt001 and Rt002 with 665 and 208 nt in length, exhibiting extremely high identity to PCV2 at the nucleotide level (99.85% and 100.00%, respectively).

### PCV2 detection and generation of the whole genome of rat-associated PCV2

**Fig. 2:**
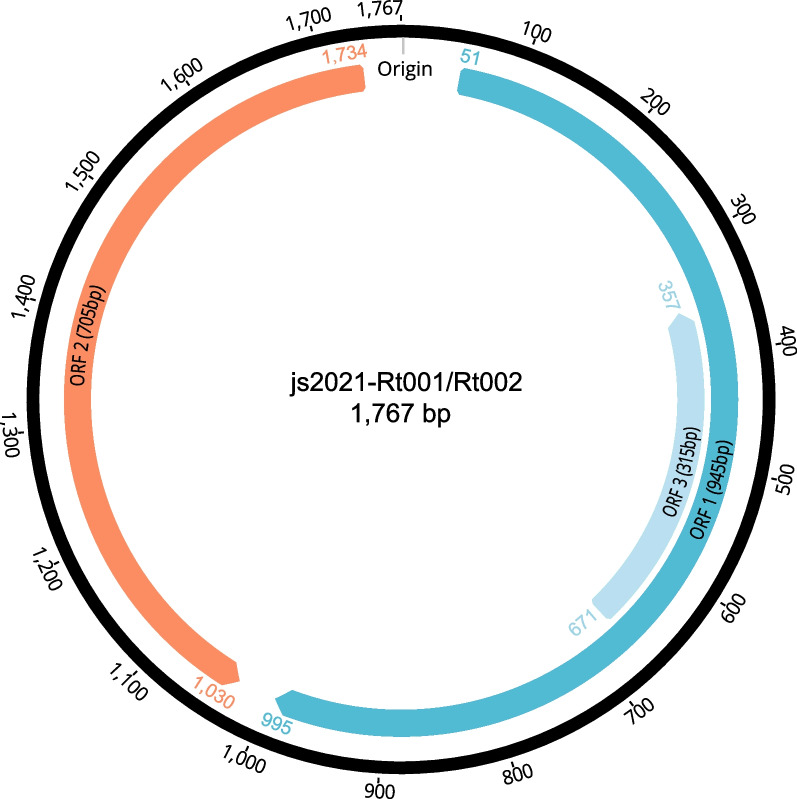
Genomic organization of the novel PCV2 viruses identified in this study

To determine the tissue distribution and genome sequence of PCV2 in the infected rats, a total of 14 nucleic acid samples were screened using the nPCR method. The PCR results showed that half of the 14 tissue samples were positive for PCV2. The positive tissue types included the kidney, heart, lung, liver, pancreas, and large and small intestines. Two distinct rat-associated PCV2 (namely js2021-Rt001 and js2021-Rt002) genomes of 1767 nt in length were amplified and sequenced successfully from positive samples from different individuals. Their G + C contents are 48.4% and 48.6%, respectively. Three open reading frames, ORF1 (945 nt), ORF2 (705 nt), and ORF3 (315 nt), in the two genomes were of the same length (Fig. 2). Pairwise-sequence alignment analysis indicated that js2021-Rt001 and js2021-Rt002 were closely related to each other, sharing 99.26% nucleotide sequence identity (13 nt differences) in full genome sequences, 99.37% (6 nt differences) in their ORF1 sequences and 99.01% (7 nt differences) in their ORF2 sequences. BLASTn analyses indicated they showed the highest nucleotide sequence identity, 99.77% and 99.55%, respectively, with the complete genome of porcine-origin PCV2 strains Han8 (GenBank no. JQ181600) and PCV2/PhuTho/G40312/2018 (GenBank no. LC602996).

### Evolutionary relationship of rat-associated PCV2

**Fig. 3:**
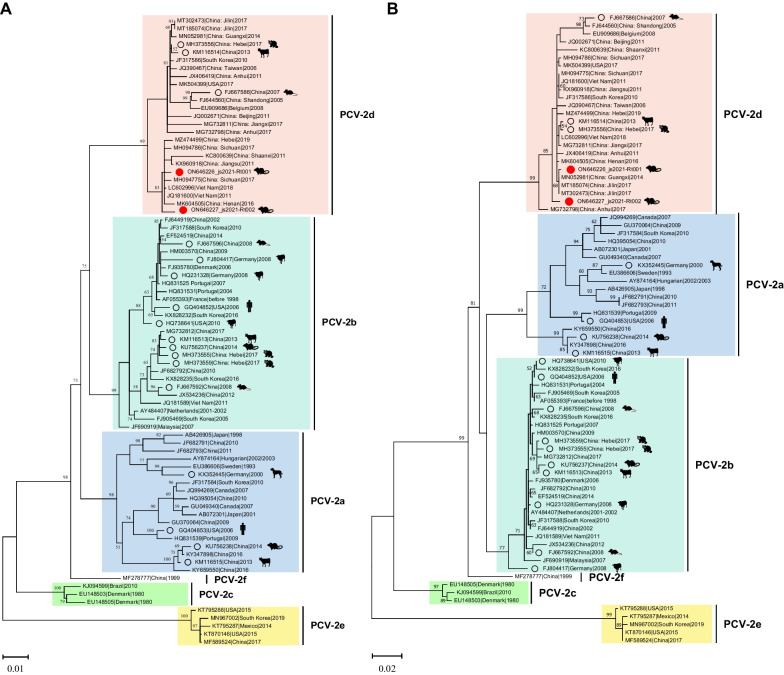
Evolutionary analyses of PCV2 using MEGA11 (v11.0.11). Maximum-likelihood (ML) trees based on nucleotide sequences of (**A**) the complete genome and (**B**) ORF2 gene of PCV2 are shown, respectively. Numbers (> 50) above or below branches are percentage bootstrap values for the associated nodes. Each scale bar represents the nucleotide substitutions per site. The newly detected rat-associated PCV2 isolates are marked with red dots. Other PCV2 sequences discovered in non-porcine hosts are pointed with black circles

A total of 77 PCV2 representative genome sequences were downloaded from the GenBank database to determine the genetic relationships of the newly discovered PCV2 strains. Owing to the competence to reconstruct the same tree as the full genome, ORF2 is also used as a phylogenetic marker for PCV2 strains. Pairwise-sequence comparisons of complete genomes and ORF2 gene sequences revealed that the nucleotide sequence identity between the two rat-associated PCV2 strains and 77 reference strains varied from 91.79% to 99.77% and 82.71% to 99.72%, respectively. Phylogenetic analyses revealed that the two complete genome sequences in Jiangsu province belonged to the recently prevalent genotype PCV2d, but js2021-Rt002 formed a monophyletic branch in both trees (Fig. 3).

### ORF2 sequence comparison

**Fig. 4:**
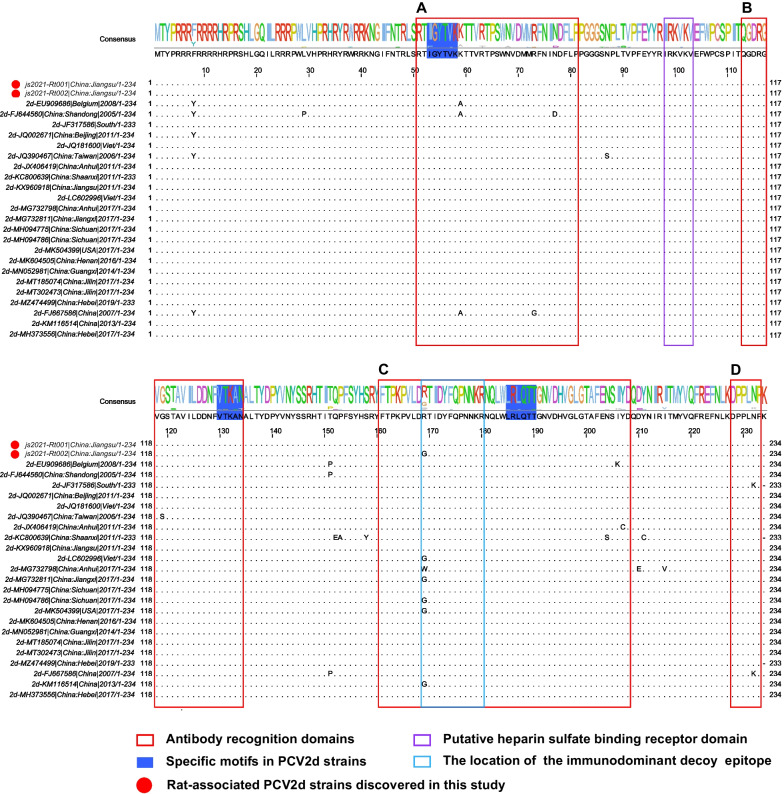
Multiple sequence alignment of ORF2 (Cap) amino acid sequences of PCV2d strains. The sequences include the novel wild rat-associated PCV2d strains (js2021-Rt001 and js2021-Rt002) and other 23 representative PCV2d strains. The blue areas in the consensus sequence show the unique motifs of PCV2d Cap sequences, which are different from other genotypes. Antibody recognition domains, heparin sulfate binding receptor domain, and immunodominant decoy epitope are shown in red, purple, and blue boxes. The strains identified in this study are indicated by red dots

Compared to amino acid sequences of other PCV2d isolates (n = 23), the 234 aa encoded by the ORF2 genes of the two novel rat-associated circoviruses were relatively conservative without any specific substitution (Fig. 4). In this study, we tried to examine the typical motifs ^53^IGYTVK^58^, ^130^VTKAN^134^, and ^185^LRLQTT^190^ for PCV2d instead of ^86^SNPLTV^91^ which is also present in PCV2c strains [39]. Consistent with previous studies, four antibody recognition domains (labeled as epitopes A–D), an immunodominant decoy epitope within epitope C, and a heparin sulfate binding motif were observed in the predicted amino acid sequences of the two rat-associated PCV2d Cap proteins [40–42]. As previously reported [41], we also identified key residues within the four epitopes: D-70, M-71, N-77 and D-78 in epitope A, Q-113, D-115 and D-127 in epitope B, Y-173, F-174, Q-175 and K-179 in epitope C, and E-203, I-206 and Y-207 in epitope D. Remarkably, there was one amino acid difference (R/G-169) in the immunodominant decoy epitope between js2021-Rt001 and js2021-Rt002.

## Discussion

Rodents rank as the largest mammalian species (approximately 43% of all mammal species). They are widely distributed and the natural reservoirs of a diverse group of pathogenic viruses [43]. In our study, the classified eukaryotic viral reads were mainly related to the genus *Picornavirales* occupying 97.31% (n = 595,373) of the total reads, while most of which were assigned to the family *Dicistroviridae* (n = 313,196) and picorna-like viruses (n = 236,984) of probable insect and environmental origin. A total of 21 viral sequences were subsequently characterized in the two rat pools after extension of contigs and nPCR amplification (Additional file 1: Table S1 and Additional file 2). Pairwise-sequence comparisons showed that these sequences shared sequence identities with their closest genetic relatives ranging from 42.2% to 99.8% at the nucleotide level, and their lengths ranged from 431 to 9787 nt. Apart from invertebrate, plant and uncultured environmental viruses, several vertebrate-infecting viral sequences were detected, including anellovirus, picornavirus, astrovirus, and retrovirus sharing > 80% nucleotide identity with previously reported viruses in rats or rat cells [44–46], together with porcine circovirus 2, known as pathogenic to pigs [47].

At present, porcine epidemic virus, PCV2, is one of the most economically important swine pathogens that has a significant impact on animal performance and production [48]. Prior to 2003, PCV2 was dominated by the PCV2a genotype [49]. On a global scale, the first genotype shift from PCV2a to PCV2b occurred around 2003 [50]. Since 2009, there has been a second genotype shift in the predominant prevalence of PCV2 [51]. Until now, PCV2d has been the predominant genotype in swine populations in China, North America, South Korea, and Uruguay [52, 53]. Since China is known for only importing swine, the reason for this genotype's global popularity remains unclear. In recent years, several studies have investigated the epidemiology of PCV2d in pigs in China: Henan, where 1283 (72.90%) of the 1760 tested samples were PCV2 positive and 47.06% (8/17) of the discovered strains belonged to PCV2d [53], Yunnan, where the percentage of PCV2 positive samples was 60.93% (170/279) and 80% (12/15) of the isolates were PCV2d [54], shanghai, in which 104 out of 199 (52.26%) were screened positive for PCV2d [52], and Jiangsu, where 34 of the 120 (28.33%) tested samples were PCV2 positive and PCV2d accounted for 47.06% of the 34 isolates [55]. The epidemiological data reveal that PCV2d has been circulating in pig-producing provinces of China for many years and recognized as a severe threat to the Chinese pig industry.

Phylogenetic trees constructed based on the full genome and ORF2 sequences showed that the two rat-associated PCV2 strains in this study belonged to the genotype PCV2d. When using js2021-Rt001 and js2021-Rt002 as query sequences, the closest hits in the BLASTn search were both the porcine-origin PCV2 isolates in Vietnam. Meantime, the detection of other genotypes in rodents inhabiting PCV2-infected pig farms [20, 30, 31] makes possible cross-species transmission of the PCV2d between porcine and rodent hosts. PCV2 ORF2 gene encodes the capsid protein, the major immunogenic protein involved in virus attachment to the host cellular receptor(s) and immune responses [40]. No aa changes were found in previously reported antibody recognition domains, an immunodominant decoy epitope, and a heparin sulfate binding motif of the rat-associated PCV2d Cap proteins [40–42]. Meantime, the ORF2 sequences of js2021-Rt001 and js2021-Rt002 were 100.00% aa identical to the Vietnam isolates, Han8 and PCV2/PhuTho/G40312/2018, respectively. Even with these evidence, the origin of the viruses remains elusive and further studies are required to confirm the potential cross-species transmission of diverse genotypes PCV2 existing between rat and porcine hosts.

It has been demonstrated that PCV2 could replicate in mice with distribution in multiple organs [26, 27, 31]. In this study, multiple tissue samples were found positive for PCV2, indicating that the two PCVs were capable of infecting the wild rats rather than only passing through the gut. Of particular note, the two highly similar PCV2 strains were present in samples collected from two wild rat individuals on different dates at the adjacent sampling sites. Horizontal and vertical transmissions were confirmed to be efficient ways for PCV2 onward spread among rodent populations [19]. This suggests the possibility of the long-term prevalence of PCV2 in the local rat populations.

PCV2 host jumps may also be a potential threat to human health. Zoonotic transmission of PCV2 has been proposed and reported in a few studies [17, 56, 57]. Rodents on swine farms have a high potential for contact with humans, posing the possibility of zoonotic transmission of PCV2 from rodents to personnel with professional occupation with pigs indirectly via contamination of water or food products. Therefore, it is necessary to capture or kill rodents on swine farms to avoid virus spread and zoonotic transmission of PCV2.

## Conclusion

In sum, to our best knowledge, this is the first report of the identified PCV2d in wild rats that were captured far from pig farms in China. This finding will help to elucidate the evolutionary relationship and epidemiology of rat-associated PCV2. But, the pathogenicity of PCV2 in rats remains unclear. More studies are needed to clarify the infectious mechanism of PCV2 in rats and the possible cross-species transmission of PCV2 between rats and pigs.

## Supplementary Information

**Additional file 1**: **Table S1**. Genomic sequences of the detected viruses

**Additional file 2**: Data on detected viral genomic sequences

## Data Availability

The sequencing raw reads analyzed in our study have been uploaded onto the Sequence Read Archive (SRA) at National Center for Biotechnology Information (NCBI) under the BioProject accession number PRJNA843194 with SRA accession numbers SRR19435143, SRR19435144, and SRR23455466 (control library). The genome sequences of js2021-Rt001 and js2021-Rt002 determined in the current study have also been deposited in GenBank under the accession numbers ON646226 and ON646227.

## Abbreviations

aa: Amino acid
dsDNA: Double-stranded DNA
dsRNA: Double-stranded RNA
ML: Maximum-likelihood
NCBI: National center for biotechnology information
NGS: Next-generation sequencing
nPCR: Nested PCR
NR: Non-redundant protein
nt: Nucleotide(s)
NT: Non-redundant nucleotide
PCV2: Porcine circovirus type 2
PCVAD: PCV-associated disease
PDNS: Porcine dermatitis and nephropathy syndrome
PMWS: Post-weaning multisystemic wasting syndrome
PRDC: Porcine respiratory disease complex
Q30: Phred quality score 30
SRA: Sequence read archive
ssDNA: Single-stranded DNA
ssRNA: Single-stranded RNA
