# Supplementary material for: Virome of wild rats (Rattus norvegicus) captured far from pig farms in Jiangsu province of China reveals novel porcine circovirus type 2d (PCV2d) sequences
Source: Virol J. 2023 Mar 9;20:46. doi: 10.1186/s12985-023-02005-2 (PMC9997004; doi:10.1186/s12985-023-02005-2)
Supplement: Supplementary file 1 — Additional file 1: Table S1. Genomic sequences of the detected viruses [file 12985_2023_2005_MOESM1_ESM.docx]

**Table S1. Data on detected viral genomic sequences.**

| **Strain** | **Organism** | **Taxonomy** | **Length (bp)** | **Hits in raw data** | **Coverage (RefSeq)** | **Coverage depth mean** | **Pairwise Identity (Best BLAST hits)** |
| --- | --- | --- | --- | --- | --- | --- | --- |
| Js2021-RtAneV | Anelloviridae sp. | *Anelloviridae; unclassified Anelloviridae* | 967 | 55 | 49.8% | 5.2 | 88.4% |
| js2021-Rt001 | Porcine circovirus 2 | *Cirlivirales; Circoviridae; Circovirus* | 1,767 | 26 | 37.6% | 2.9 | 99.8% |
| js2021-Rt002 | Porcine circovirus 2 | *Cirlivirales; Circoviridae; Circovirus* | 1,767 | 2 | 11.8% | 0.2 | 99.6% |
| Js2021-RtGenoV001 | Rat-associated gemycircularvirus | *Geplafuvirales; Genomoviridae; Gemycircularvirus* | 2,252 | 64 | 100.0% | 6.5 | 99.4% |
| Js2021-RtGenoV002 | Rat-associated genomovirus | *Geplafuvirales; Genomoviridae; unclassified Genomoviridae* | 1,515 | 39 | 80.6% | 3.8 | 98.6% |
| Js2021-RtssDNAV | Rat-associated circular DNA virus | *Cressdnaviricota; unclassified Cressdnaviricota* | 1,158 | 98 | 100.0% | 17.7 | 42.2% |
| Js2021-RtDicV001 | Dicistroviridae sp. | *Picornavirales; Dicistroviridae; unclassified Dicistroviridae* | 9,072 | 2,078 | 100.0% | 55.4 | 52.6% |
| Js2021-RtDicV002 | Dicistroviridae sp. | *Picornavirales; Dicistroviridae; unclassified Dicistroviridae* | 8,728 | 96,528 | 100.0% | 2715.2 | 49.3% |
| Js2021-RtRaboV | Rabovirus A | *Picornavirales; Picornaviridae; Rabovirus* | 7,777 | 10,898 | 100.0% | 325.0 | 83.9% |
| Js2021-RtRosaV | Rosavirus B | *Picornavirales; Picornaviridae; Rosavirus* | 8,960 | 21,733 | 100.0% | 571.2 | 90.8% |
| Js2021-RtPicoLV001 | Rat-associated picorna-like virus | *Picornavirales; unclassified Picornavirales* | 9,000 | 1,174 | 100.0% | 29.6 | 90.8% |
| Js2021-RtPicoLV002 | Rat-associated picorna-like virus | *Picornavirales; unclassified Picornavirales* | 9,787 | 7,344 | 100.0% | 167.4 | 97.7% |
| Js2021-RtPicoLV003 | Rat-associated picorna-like virus | *Picornavirales; unclassified Picornavirales* | 9,379 | 6,687 | 100.0% | 164.7 | 48.5% |
| Bastrovirus/CHN/Rat/77 (Ref-Seq) | Bastrovirus Rat/77 (Ref-Seq) | *Stellavirales; Astroviridae; unclassified Astroviridae* | 5,918 | 33 | 40.8% | 1.3 | 93.9% |
| Js2021-RtVirgaV | Pepper mild mottle virus | *Martellivirales; Virgaviridae; Tobamovirus* | 4,970 | 314 | 95.8% | 12.2 | 99.4% |
| Js2021-RtSoleV | Solemoviridae sp. | *Sobelivirales; Solemoviridae; unclassified Solemoviridae* | 3,077 | 223 | 100.0% | 16.7 | 76.4% |
| Js2021-RtRibo001 | Riboviria sp. | *Riboviria; unclassified Riboviria* | 4,723 | 1,686 | 100.0% | 86.5 | 43.8% |
| Js2021-RtRibo002 | Riboviria sp. | *Riboviria; unclassified Riboviria* | 3,494 | 1,837 | 100.0% | 126.1 | 53.1% |
| Js2021-RtChuV segment1 | Rat-associated chuvirus-like virus | *Jingchuvirales; Chuviridae; Scarabeuvirus* | 6,936 | 969 | 100.0% | 33.9 | 47.8% |
| Js2021-RtChuV segment2 | Rat-associated chuvirus-like virus | *Jingchuvirales; Chuviridae; Scarabeuvirus* | 4,341 | 3,361 | 100.0% | 185.0 | 45.3% |
| Js2021-RtRTV | Rat retrovirus | *Ortervirales; Retroviridae; Orthoretrovirinae; Gammaretrovirus* | 431 | 8 | 22.0% | 0.4 | 98.6% |
